# Climate-related environmental stress in intertidal grazers: scaling-up biochemical responses to assemblage-level processes

**DOI:** 10.7717/peerj.2533

**Published:** 2016-10-20

**Authors:** Elena Maggi, Mario Cappiello, Antonella Del Corso, Francesca Lenzarini, Eleonora Peroni, Lisandro Benedetti-Cecchi

**Affiliations:** 1 Department of Biology, University of Pisa, Pisa, Italy; 2 CoNISMa, Consorzio Nazionale Interuniversitario per le Scienze del Mare, Roma, Italy

**Keywords:** Extreme climatic events, Rocky intertidal gastropod, Antioxidant mechanisms, Phenotypic plasticity, Ecological scaling, Interaction strength

## Abstract

**Background:**

Organisms are facing increasing levels of environmental stress under climate change that may severely affect the functioning of biological systems at different levels of organization. Growing evidence suggests that reduction in body size is a universal response of organisms to global warming. However, a clear understanding of whether extreme climate events will impose selection directly on phenotypic plastic responses and how these responses affect ecological interactions has remained elusive.

**Methods:**

We experimentally investigated the effects of extreme desiccation events on antioxidant defense mechanisms of a rocky intertidal gastropod (*Patella ulyssiponensis*), and evaluated how these effects scaled-up at the population and assemblage levels.

**Results:**

With increasing levels of desiccation stress, limpets showed significant lower levels of total glutathione, tended to grow less and had reduced per capita interaction strength on their resources.

**Discussion:**

Results suggested that phenotypic plasticity (i.e., reduction in adults’ body size) allowed buffering biochemical responses to stress to scale-up at the assemblage level. Unveiling the linkages among different levels of biological organization is key to develop indicators that can anticipate large-scale ecological impacts of climate change.

## Introduction

There is increasing concern among scientists, policy-makers and the general public about the societal and environmental consequences of climate change. Climate events can affect society directly by causing catastrophes and by threatening human health and indirectly by reducing environmental quality (Easterling et al., 2000). Recent models agree that the frequency, intensity and duration of extreme events such as heat waves, droughts and storms are increasing with climate change (Intergovernmental Panel on Climate Change, 2013). With extreme climate events becoming more likely, living organisms are facing increasing levels of environmental stress that may severely affect the functioning of biological systems at different levels of organization (Easterling et al., 2000). Reduction in body size has been proposed as a third universal response of organisms to global warming (Daufresne, Langfellner & Sommer, 2009), in addition to shifts in species’ distributional ranges and changes in phenology (Visser & Both, 2005; Durant et al., 2007). Recent reviews concluded that evidence for genetic adaptation is scarce, and observed patterns are mostly the result of phenotypic plasticity (Merilä & Hendry, 2014; Reusch, 2014). An important and still unresolved question is whether climate-related increases in environmental extremes and fluctuations will impose selection directly on plasticity (Pigliucci, 2005; Chevin, Collins & Lefevre, 2013).

When environmental changes translate into increased stress levels at the edge of species tolerance ranges (such as during extreme events), selection is for enhanced tolerance. This is called ‘phenotypic buffering’, a special case of plasticity (Reusch, 2014). Examples of phenotypic buffering include the activation of specific enzymes, production of water soluble reductants and enhanced expression of proteins (e.g., shock proteins in response to heat stress), which are important to maintain proper levels of cellular metabolism (Abele & Puntaruolo, 2004). Indeed, one of the most common adverse effects of extreme environmental conditions on living organisms is an increase in oxidative stress (Paital et al., 2016) that can be balanced by a number of antioxidant defenses, both enzymatic and non-enzymatic (for a review see Birben et al., 2012). A first line of defense against oxidative stress is exerted by a number of low molecular weight molecules (e.g., glutathione, ascorbate, thioredoxin) that can react directly with reactive oxygen species (ROS) and their oxidation products. In turn, enzymes can counteract oxidative stress directly, transforming ROS into less toxic molecules (e.g., catalase and superoxide dismutase) or indirectly, preserving pools of molecules that can react with ROS (e.g., glutathione reductase). Many studies have demonstrated negative effects of extremities in climate-related variables at a variety of biological levels, from DNA, proteins to abundance and distribution of assemblages (e.g., Benedetti-Cecchi et al., 2006; Callegari & Kelly, 2006; Kawai-Yamada et al., 2005; Grant et al., 2014). However, to the best of our knowledge, no study has investigated how effects at sub-cellular, cellular and organism levels scale-up to natural populations and ecological communities.

Intertidal animals are exposed to variable physical conditions that can easily become detrimental to their life and this variability is increasing with extreme climate events becoming more likely (Benedetti-Cecchi, 2003; Benedetti-Cecchi et al., 2006; Harley et al., 2006; Maggi et al., 2012). In particular, predicted changes in intensity and duration of temperature extremes (Intergovernmental Panel on Climate Change, 2013) will increase the likelihood of extreme desiccation events, due to prolonged aerial exposure and temperature increase (Houghton et al., 2001), especially in micro-tidal systems such as the northwest Mediterranean (Benedetti-Cecchi et al., 2006). Extreme desiccation can affect individual physiology (with sublethal and lethal consequences; Tsuchiya, 1983; Helmuth & Hofmann, 2001; Silva et al., 2005), but also population phenotypic traits, such as growth rates (Jones & Boulding, 1999). Intertidal molluscs appear particularly vulnerable to global warming (Hawkins et al., 2009), and warming-related changes in phenology has been observed in both reproductive and morphological traits (Moore, Thompson & Hawkins, 2011; Petes, Murphy & Menge, 2007). We experimentally investigated the effects of increasing desiccation stress on antioxidant defense mechanisms (specifically concentrations of total glutathione, catalase and glutathione reductase) of a rocky intertidal gastropod grazer (namely *Patella ulyssiponensis*), and evaluated if these effects scale-up to population and assemblage levels through changes in a phenotypic trait (i.e., their growth rate) and through changes in the interaction strength between the grazer and its resources.

## Material & Methods

### Study system

The study was carried out along the rocky coast south of Livorno (Italy, Western Mediterranean Sea; 43°28^′^02N, 10°22^′^19E) from March to May 2009.

Mediterranean rocky intertidal habitats are extremely variable environments due to the limited amplitude of tides. Barometric pressure is the primary force determining the position of the sea level, largely dictating the timing and duration of aerial exposure of organisms along the intertidal gradient. Changes in barometric pressure can therefore induce abrupt fluctuations in thermal conditions, particularly in the high-shore habitat, where temperature in Spring can vary over a range of more than 15°C and reach values up to 30°C (Benedetti-Cecchi et al., 2006).

Differences among algal assemblages at different heights on the shore are mainly due to changes in relative abundance of species. Low-shore assemblages are dominated by encrusting, filamentous and coarsely branched algae. In contrast, barnacles (mainly *Chthamalus stellatus* (Poli), but also *C. montagui* (Southward)) and cyanobacteria (*Rivularia* spp.) dominate the high-shore (Benedetti-Cecchi, 2000; Benedetti-Cecchi, 2001). Among most common grazers, *Patella ulyssiponensis* (Gmelin) is characterized by a relatively wide vertical range of distribution (from mid/high- to low-shore habitats). It is a generalist herbivore (Della Santina et al., 1993), feeding either on small-sized macroalgae, such as filamentous ones, or on the microscopic components of biofilm colonizing apparent bare rock at all heights (cyanobacteria, diatoms or macroalgal spores). A recent study indicated that Mediterranean limpets already exist on the edges of their thermal tolerance windows. In particular, *P. ulyssiponensis*, although characterized by a low metabolism, is very sensitive to exposure to elevated temperatures (Prusina et al., 2014), as those naturally experienced at the study site (Benedetti-Cecchi et al., 2006).

### Experimental design and field sampling

We imposed increasing levels of desiccation stress by confining individuals of *P. ulyssiponensis* at increasing heights on the shore within their natural range of vertical distribution (from 0 (low-shore) to 15 (mid-shore) or 30 cm (high-shore) above MLWL), by means of 17 × 17 cm fenced plots. By precluding limpets to move down the shore for a period of about two months, we simulated a scenario of extreme thermal stress that can be expected under climate change. The *in situ* estimated difference in daily average temperature between low- and high-shore for the study period was 1.3°C (Benedetti-Cecchi et al., 2006), within the range of expected increases in extreme surface air temperature for the period 2016–2035 (Intergovernmental Panel on Climate Change, 2013). For each height on the shore, three replicate fenced plots were positioned within each of three areas (stretches of coast extending about 3 m alongshore and 10 s of meters apart). Limpets were taken from the low-shore with blunt metal sheets to avoid breaking the shell and were immediately transplanted to plots (*n* = 2 limpets per plot) by gently pressuring them against the rock for some minutes to facilitate their attachment. Each individual was marked with blue epoxy putty (Subcoat S, Venziani) either in the centre or at the margin of the shell, for subsequent identification. Length of the major axis of the shell was measured by means of a plastic calliper (size range: 13–23 mm, length of major axis of the shell). To control for possible artefacts associated with the manipulation of grazers, additional pairs of limpets were detached, marked and placed back to their original position from each of 3 open plots (17 × 17 cm) within each of 3 additional areas in the low-shore habitat. Occasionally missing individuals from fenced plots were replaced by new ones to maintain approximately constant limpet density during the experiment.

At the end of the experiment, marked limpets were collected, measured, and maintained at −80°C for subsequent analysis. Percentage cover of filamentous algae (their main macroscopic resource) within plots was visually estimated with a plastic frame of 15 × 15 cm, divided into 16 sub-quadrats. A score from 0 (absence) to 4 (full cover) was given in each sub-quadrat and a final estimate was obtained by summing individual scores over the 16 sub-quadrats (Dethier et al., 1993), for a maximum score of 64. Values were then expressed as percentages.

### Biochemical analyses

Frozen limpets were shelled, weighed, cut in small pieces and homogenized with a hand-driven Potter Elvejhem glass homogenizer in 5 vol of 50 mM Tris HCl (pH 7.4). The homogenate was centrifuged in a Millifuge centrifuge (Millipore) for 30 s to remove debris. The supernatant (crude extract) was divided into two aliquots. An aliquot was used without further treatment for the assay of catalase and glutathione reductase; the other aliquot was immediately acidified to pH 2 by the addition of 4 N HCl in order to preserve glutathione in the extract, centrifuged again and used for the measurement of glutathione concentration.

The activity of catalase was measured at 25°C in accordance with Luck (1965), by following the decrease in absorbance at 240 nm (Δε = 0.0436 mM^−1^ cm^−1^). The reaction mixture contained, in 50 mM sodium phosphate buffer (pH 7.4), 12.5 mM hydrogen peroxide.

The activity of glutathione reductase was determined at 30°C in accordance with Racker (1955) by following the decrease in absorbance at 340 nm (Δε = 6.22 mM^−1^ cm^−1^). The reaction mixture contained, in 160 mM potassium phosphate buffer (pH 7.4), 0.5 mM oxidized glutathione, 0.11 mM NADPH and 1 mM EDTA. The activities of catalase and glutathione reductase were normalized for protein concentration.

**Table 1:** Mixed-effect models on total glutathione, catalase and glutathione reductase concentrations, and growth rate of limpets from low-shore habitat. Limpets from fences were compared to limpets marked and disturbed (PC, procedural control) in open plots. A total of three fences or open plots were deployed within each of three random areas.

		Total glutathione	Catalase	Glutathione reductase	Growth rate
Fixed effects	Coefficient (SE)				
Intercept	*γ*_00_	0.773 (0.111)^***^	33.849 (6.769)^***^	0.007 (0.001)^***^	17.86 (0.552)^***^
Fences *vs.* PC	*γ*_01_	−0.142 (0.159)	−3.675 (9.975)	0.001 (0.001)	−0.283 (0.810)
Random effects	Variances (SD)				
Area	${\sigma }_{Area}^{2}$	0.000 (0.000)	8.141 (2.853)	0.000 (0.000)	0.000 (0.000)
Plot(Area)	${\sigma }_{Plot}^{2}$	0.082 (0.287)	282.609 (16.811)	0.000 (0.001)	0.000 (0.000)
Residual	${\sigma }_{e}^{2}$	0.065 (0.256)	377.246 (19.423)	0.000 (0.002)	4.922 (2.218)

**Notes.**

*** *p* < 0.001.

Total glutathione concentration was measured by using a colorimetric end point coupled enzymatic assay (Cappiello et al., 2013), based on the measurement of cysteine produced from glutathione by *γ*-glutamyltransferase and leucyl aminopeptidase. Briefly, the standard incubation mixture (250 µL final volume) contained 8 mM MgCl_2_, 0.2 mM MnCl_2,_2 mM dithiothreitol, 40 mM Gly-Gly, 50 mU/mL *γ*-glutamyltransferase and 50 mU/mL leucyl aminopeptidase in 32 mM Tris HCl pH 8.5. The reaction was started by the addition of limpet extracts. After 30-min incubation at 37°C, the reaction was stopped with the addition of 12.5 µL of 100% (w/v) trichloroacetic acid and the incubation mixture was centrifuged at 12,000 ×g for 1 min in a Beckman Microfuge E. The cysteine formed was then evaluated spectrophotometrically. An aliquot of 200 µL of the supernatant was added to 200 µL of glacial acetic acid and 200 µL of a reagent, prepared by dissolving 250 mg of ninhydrin in 10 mL of glacial acetic acid/4 M HCl (3:2). The mixture was placed in a boiling bath for 4 min. Under these conditions, cysteine specifically reacted with ninhydrin to give a pink colored complex. After cooling on ice, 300 µL of the mixture were diluted with 300 µL of 95% ethanol and the absorbance at 560 nm measured. A reference standard curve constructed with known concentrations of cysteine was used for the detemination of cysteine in samples.

Protein concentration was estimated by the Coomassie Blue binding assay (Bradford, 1976), with bovine serum albumin as the standard. Total glutathione concentration was normalized for wet weight of each individual limpet analyzed.

### Data analyses

Values of catalase, glutathione reductase, total glutathione concentration and growth rate of limpets were analysed by means of mixed-effect models. Growth rate was calculated as (Length_*FINAL*_−Length_*INITIAL*_)/ Length_*INITIAL*_. Areas and plots nested in areas were included as random effects into the model. A first set of analyses examined possible artefacts associated with the experimental procedure, by comparing limpets from fenced plots in the low-shore habitat and those relocated inside or near open plots at the same height on the shore (i.e., marked and disturbed in the low-shore habitat; PC, procedural control) (Fences *vs.* PC, fixed effect). As no significant difference emerged for any response variable (Table 1), these replicates were merged into a single low-shore level treatment, to increase sample size. Data were then analysed by including height on the shore as a continuous predictor variable (i.e., with the low-, mid- and high-shore treatments corresponding to values of 0, 15 and 30 (cm above MLWL), respectively) (Height, fixed effect), (Table 2). The newly introduced limpets compensating for missing ones remained exposed to experimental conditions for shorter periods than the original animals. To control for this effect, the previous analysis was also performed by including duration to treatment conditions as a covariate (Permanence, fixed effect). For each variable, this new model was compared to the reduced model without the covariate, by means of a log-likelihood ratio test. The effect of the covariate was never significant, nor did it significantly increase the goodness of fit of any of the models examined (Table 3). Analyses were run using the “lmer” function (able to deal with unbalanced data) from “lme4” package (R v2.15.3; R Development Core Team, 2013). Normality and homoscedasticity of data was assessed by normal QQ-plots and by plotting residuals *vs.* fitted values.

**Table 2:** Mixed-effect models on total glutathione, catalase and glutathione reductase concentrations, and growth rate of limpets at different heights on the shore (low-, mid- or high-shore). For each height on the shore, three plots (=fences) were deployed within each of three random areas. For the low-shore habitat, limpets from three additional plots (=open plots, PC) within each of three random areas were included in the analyses.

		Total glutathione	Catalase	Glutathione reductase	Growth rate
Fixed effects	Coefficient (SE)				
Intercept	*γ*_00_	0.707 (0.066)^***^	29.623 (4.301)^***^	0.007 (0.001)^***^	17.363 (0.449)^***^
Height	*γ*_01_	−0.008 (0.004)^*^	−0.043 (0.263)	−0.000 (0.000)	−0.015 (0.026)
Random effects	Variances (SD)				
Area	${\sigma }_{Area}^{2}$	0.000 (0.000)	49.09 (7.006)	0.000 (0.000)	0.470 (0.685)
Plot(Area)	${\sigma }_{Plot}^{2}$	0.058 (0.242)	0.00 (0.00)	0.000 (0.001)	0.383 (0.619)
Residual	${\sigma }_{e}^{2}$	0.047 (0.217)	520.85 (22.822)	0.000 (0.003)	4.444 (2.108)

**Notes.**

* <0.05.

*** *p* < 0.001.

**Table 3:** Mixed-effect models on total glutathione, catalase and glutathione reductase concentrations, and growth rate of limpets at different heights on the shore (low-, mid- or high-shore). To control for the effect of newly introduced limpets compensating for missing ones, duration to treatment conditions of individual limpets was included as a covariate (Permanence). For each height on the shore, three plots (=fences) were deployed within each of three random areas. For the low-shore habitat, limpets from three additional plots (=open plots, PC) within each of three random areas were included in the analyses. For each variable, the model was compared to the reduced model without the covariate (see Table 2), by means of a log-likelihood ratio test.

		Total glutathione	Catalase	Glutathione reductase	Growth rate
Fixed effects	Coefficient (SE)				
Intercept	*γ*_00_	0.384 (0.244)	14.957 (17.447)	0.009 (0.002)^***^	0.006 (0.053)
Height	*γ*_01_	−0.008 (0.004)^*^	−0.059 (0.293)	−0.000 (0.000)	−0.002 (0.001)
Permanence	*γ*_02_	0.005 (0.004)	0.242 (0.278)	−0.000 (0.000)	0.001 (0.001)
Random effects	Variances (SD)				
Area	${\sigma }_{Area}^{2}$	0.000 (0.000)	88.36 (9.40)	0.000 (0.000)	0.000 (0.000)
Plot(Area)	${\sigma }_{Plot}^{2}$	0.055 (0.235)	0.000 (0.000)	0.000 (0.000)	0.003 (0.053)
Residual	${\sigma }_{e}^{2}$	0.047 (0.218)	502.69 (22.42)	0.000 (0.003)	0.006 (0.079)
LogLik test	*χ*^2^	1.182	0.607	0.739	1.138

**Notes.**

* <0.05.

*** *p* < 0.001.

An index of ‘per capita interaction strength’ quantifying the effect of limpets on their macroscopic resources (filamentous algae) was calculated for each height on the shore. As *P. ulyssiponensis* is generalist species, we assumed the consumption of filamentous algae was a good estimate of the general feeding activity of these limpets. We used a modified version of the index proposed by Paine (1992). In the original formula, the relative change in abundance of a resource in presence or absence of a consumer is standardized against consumer density. In our case, the index was calculated by comparing enclosure and natural plots, separately for each height on the shore. Our study system, however, is characterized by an extremely patchy distribution of algae and limpets, particularly in the high-shore habitat, making estimates of mean consumption rates from natural plots quite challenging. To take into account this feature, we opted for using long term data from ancillary studies conducted at the same site, to derive expectations of the mean values of abundance of the resource and density of consumer in control plots (*C* and *d*_*C*_, respectively) at each tidal height. We then calculated the relative change in percentage cover of filamentous algae between enclosures (*E*) and natural plots in the surrounding environment (*C*), divided by the difference in density of grazers between the two conditions (*d*_*E*_, equal to 2 individuals of *P. ulyssiponensis* in each fence, and *d*_*C*_, lower than 2 individuals at all heights):

\begin{eqnarray*} \frac{E-C}{C({d}_{E}-{d}_{C})} \end{eqnarray*}

The lower the interaction strength, the greater the impact of an individual limpet within enclosures compared to the surrounding open rock surface. Mean estimates and 95% confidence intervals of per capita effects were estimated by bootstrapping the original data 10,000 times separately for each tidal height. Analysis was run using the “boot” package (R v2.15.3, R Development Core Team, 2013).

## Results

**Figure 1:**
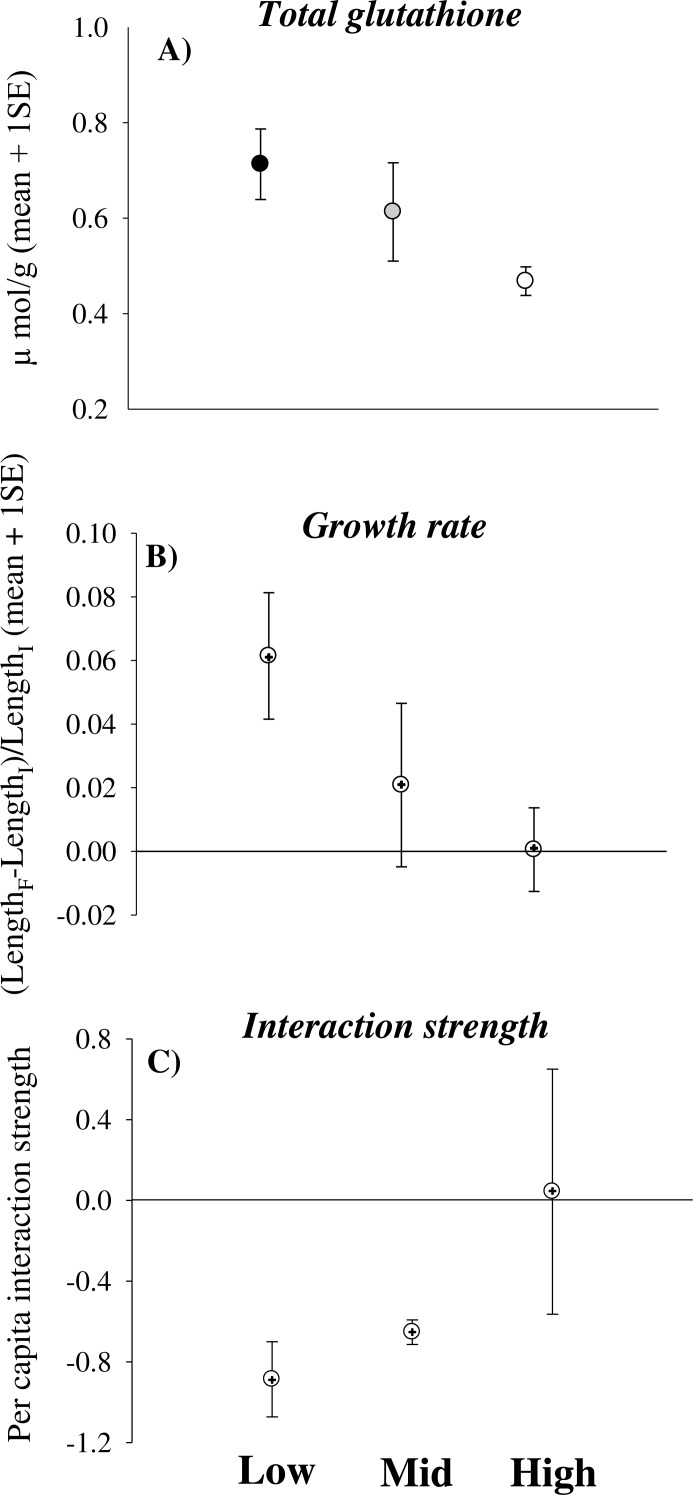
Physiological effects of desiccation stress scale-up to the population and assemblage levels in rocky intertidal limpets. (A) Total glutathione concentration, (B) growth rate and (C) per capita interaction strength for limpets exposed to increasing levels of desiccation stress (the lower the interaction strength, the greater the impact of limpets within enclosures compared to the surrounding open rock surface). In (A) data are means and sample standard errors (sample size was 28 individuals for the low-shore and ranged between 11 and 14 for the mid- and high-shore). In (B) and (C) data are bootstrapped means ± 95% CIs, as well as mean values from observed data (+).

Total glutathione significantly decreased in limpets experiencing increasingly higher desiccation stress [Height, coefficient (se) = −0.008 (0.004), *p* < 0.05] (Fig. 1A). In contrast, catalase and glutathione reductase did not change significantly with increasing height on the shore (Table 2). Some animals underwent negative growth (shrinking) and this generated some variation in growth data. Nevertheless, the analysis showed a trend of decreasing growth rates of limpets with increasing height on the shore [Height, coefficient (se) = −0.015 (0.026), 0.05 < *p* < 0.1] (Table 3 and Fig. 1B). Indeed, shrinking was observed on 40% of individuals from mid- and high-shore but only on 10% of individuals from low-shore habitat. Per capita effects of grazing limpets on filamentous algae decreased with increasing desiccation stress. In particular, mean bootstrapped values of the index changed from negative in the low-shore habitat to zero under high desiccation stress in the high-shore habitat (Fig. 1C).

## Discussion

Limpets experiencing high desiccation stress showed significant lower levels of total glutathione, tended to grow less and had reduced per capita interaction strength on their main macroscopic resource in comparison to individuals living in a more benign environment. In case of extreme events, the most likely processes for maintaining a functional phenotype in aerobic organisms are buffering responses (Reusch, 2014), such as increased production of reactive oxygen species through activation and consumption of water soluble reductants (Abele & Puntaruolo, 2004). In our experiment, animals showed decreasing levels of total glutathione at increasing desiccation stress. Glutathione is a small antioxidant molecule, whose reduced form is oxidized in presence of oxygen free radicals. Reduced concentrations of glutathione have been observed in marine invertebrates exposed to thermal stress, such as mussels and sponges (Bachinski et al., 1997; Bocchetti & Regoli, 2006; Falfushynska et al., 2014). Lack of a concomitant decrease in antioxidant enzymes, such as catalase and glutathione reductase, suggests that limpets did not cross the critical temperature at which anaerobic metabolism is activated to prolong passive survival (Abele & Puntaruolo, 2004), when they lose the ability to activate enzyme defences. However, desiccation stress was intense enough to influence the amount of energy and metabolic capacity available for fitness-related functions, such as feeding and growth (Pörtner & Farrell, 2008).

In natural populations, changes in environmental conditions can be easily accompanied by morphological and life-history trait variations among individuals (Stenseth et al., 2002; Walther et al., 2002). Such traits are often ‘labile,’ that is they can be expressed repeatedly across the lifetime of individuals and can vary over the ontogenetic course of each organisms. Examples include reproductive traits, morphological characters and measures of body size. When this plasticity allows organisms to vary their optimal phenotype, the evolution of an adaptation is predicted (Nussey, Wilson & Brommer, 2007). In particular, theory predicts a selection for smaller body size under global warming, especially in aquatic systems, due to an increase in the proportion of small-sized species and young age classes, as well as a reduced size at maturity (Forster, Hirst & Atkinson, 2012). These responses are expected under moderate long-term increases in warming-related stresses and are interpreted as phenotypic responses, but evolutionary adaptations could also take place, through selection of traits promoting plasticity. On the contrary, the duration of extreme events is considered too short so that only phenotypic buffering mechanisms can take place (Reusch, 2014). Our results, however, suggested that aquatic ectotherms might respond with a reduction in adults’ growth rate also to short-term stress events, by readily scaling-up a biochemical buffering response through a true phenotypic response. This is of major importance, as plastic responses to extreme climate events may keep populations above a critical threshold until adaptive evolution has improved mean population fitness (Lande, 2009), thus highlighting the need to incorporate phenotypic plasticity into ‘evolutionary rescue’ approaches (Reusch, 2014).

In addition, such phenotypic responses may have profound implications at the ecosystem scale, as reduction in body size may affect the intensity of trophic interactions through cascading effects (Daufresne, Langfellner & Sommer, 2009). Results from the present study corroborated theses expectations, by showing a decrease in the per capita interaction strength between limpets and filamentous algae at increasing desiccation stress. This would suggest that grazers dealt with extreme environmental conditions by lessening their feeding pressure on macroscopic resources, probably as a consequence of reduced growth rate (and so energy requirements), which in turn was negatively affected by the reduction in resource uptake. However, the between-individual variability in the response to extreme climatic events was quite high at the trophic interaction level, especially in comparison to the biochemical and morphological responses. Indeed, the concentration of total glutathione decreased quite homogeneously among individuals at increasing desiccation stress and a relatively low variability was observed in the trait response related to body size. On the contrary, the large confidence intervals around the trophic strength of limpets on macroalgae might suggest that some individuals, despite reducing their growth rate, were still able to invest energy in feeding activities. This could be of major importance, for example, for enabling organisms to maintain other fitness related traits, such as high reproductive rates, under highly stressful conditions.

The ability of populations to deal with climate changes by means of between-individual variation in plastic responses is of wide interest for evolutionary ecologists. During the last decade, this interest has stimulated the use of analytical frameworks based, for example, on the reaction norm concept, to examine the causes and consequences of variation in life history plasticity in the wild (Nussey, Wilson & Brommer, 2007; Reusch, 2014; Taff & Vitousek, 2016). The application of these promising tools could add important information about mechanisms involved and possible consequences of a scaling-up of biochemical responses to the ecosystem level, under a dynamic world.

To the best of our knowledge, the present study is the first attempt to investigate the mechanisms involved in the scaling-up of biochemical responses to assemblage-level processes. Unveiling the linkages among different levels of biological organization could be key to develop cost-effective indicators of change that can reliably anticipate large-scale biological consequences of climate extremes.

##  Supplemental Information

10.7717/peerj.2533/supp-1 Data S1 Mean values of concentration of total glutathione, catalase and glutathione reductase, of percent growth rate and interaction strength index for each experimental plot

## Additional Information and Declarations

### Data Availability

The following information was supplied regarding data availability: The raw data has been supplied as Data S1.
